# Inflammatory Mechanisms of Diabetes and Its Vascular Complications

**DOI:** 10.3390/biomedicines10051168

**Published:** 2022-05-18

**Authors:** Lyudmila V. Nedosugova, Yuliya V. Markina, Leyla A. Bochkareva, Irina A. Kuzina, Nina A. Petunina, Irina Y. Yudina, Tatiana V. Kirichenko

**Affiliations:** 1 Sechenov First Moscow State Medical University (Sechenov University), 119991 Moscow, Russia; profmila@mail.ru (L.V.N.); lejlani@mail.ru (L.A.B.); mia986@mail.ru (I.A.K.); urleur@yandex.ru (N.A.P.); mamaikozlovskaya@gmail.com (I.Y.Y.); 2 Petrovsky National Research Center of Surgery, 119991 Moscow, Russia; yu.v.markina@gmail.com; 3 Chazov National Medical Research Center of Cardiology, 121552 Moscow, Russia

**Keywords:** diabetes, inflammation, cytokines, macrophages, mitochondria, insulin resistance

## Abstract

The main cause of death in patients with type 2 DM is cardiovascular complications resulting from the progression of atherosclerosis. The pathophysiology of the association between diabetes and its vascular complications is complex and multifactorial and closely related to the toxic effects of hyperglycemia that causes increased generation of reactive oxygen species and promotes the secretion of pro-inflammatory cytokines. Subsequent oxidative stress and inflammation are major factors of the progression of type 2 DM and its vascular complications. Data on the pathogenesis of the development of type 2 DM and associated cardiovascular diseases, in particular atherosclerosis, open up broad prospects for the further development of new diagnostic and therapeutic approaches.

## 1. Introduction

The prevalence of the chronic progressive disease diabetes mellitus is growing steadily every year all over the world. According to International Diabetes Federation, there are 537 million adults with diabetes in the world today. Moreover, according to current predictions, this number is expected to exceed 643 million by 2030 and 780 million by 2045 [1]. About 10% of patients have type 1 diabetes, and the rest have type 2 diabetes mellitus (DM). In addition, for every patient diagnosed with type 2 DM, there is one person with an undiagnosed disease. Therefore, the real number of patients with diabetes is much higher. Mortality from diabetes in 2021 exceeded 6.7 million people, which is more than the total mortality from AIDS, tuberculosis, and malaria [1]. There is no doubt that diabetes is one of the most significant risk factors for severe COVID-19, with increased admissions to intensive care unit and mortality among coronavirus patients [2,3]. The main cause of death in patients with type 2 DM is cardiovascular complications resulting from the progression of atherosclerosis. Several studies have shown a higher incidence of cardiovascular diseases (coronary heart disease, heart failure, etc.) in patients with diabetes mellitus compared with people of the same age without diabetes [4]. Despite the obvious advances in the treatment of cardiovascular diseases, type 2 DM has a significant impact on their prognosis. The risk of diabetic macroangiopathy development increases with elevation of glycemic level, indicating an almost linear relationship between metabolic disorders in type 2 DM and vascular damage [5,6]. According to modern concepts, hyperglycemia activates glucose autooxidation and contributes to a decrease in the activity of antioxidant defense, causing excessive accumulation of free radicals, which leads to the development of oxidative stress. It seems possible to reduce the severity of oxidative stress and, accordingly, to ameliorate the progression of diabetic vascular complications, improving the prognosis and quality of life of patients with type 2 DM by eliminating the negative effect of hyperglycemia through adequate hypoglycemic therapy. However, the pathophysiology of the association between diabetes and vascular complications is complex and multifactorial. In fact, the mechanism of the pathogenesis of cardiovascular disease in type 2 DM is not fully understood, but it appears to be closely related to the toxic effects caused by hyperglycemia as well as obesity-induced hyperlipidemia–gluco- and lipotoxicity, respectively. Hyperglycemia causes oxidative stress due to mitochondrial dysfunction and increased generation of reactive oxygen species (ROS), while hyperlipidemia promotes adipose tissue expansion that activates Map kinases and the release of cytokines which come from a change in immune cell phenotype. Subsequent oxidative stress and moderate inflammation are major factors in the progression of type 2 DM vascular complications [7,8].

## 2. Inflammation in Insulin Resistance and Diabetes Mellitus

Inflammation is seen as a critical factor in metabolic dysregulation. Suppression of inflammatory reactions has been considered a metabolically protective process that reduces the development of insulin resistance and type 2 DM. Over a century ago, high doses of sodium salicylate were found to reduce glucosuria in people diagnosed with diabetes [9,10]. Insulin resistance of the liver, adipose tissue, and skeletal muscle stimulates the secretion of insulin from the pancreas, which maintains a normal level of glycemia (pre-diabetic stage) [11,12,13]. The effective relationship between insulin-secreting cells and insulin target tissues (pancreas, adipose tissue, liver, and skeletal muscle) maintains metabolic homeostasis in response to physiological fluctuations in glycemia or lipemia, in response to food intake or starvation. Insulin resistance represents a partial disruption of communication between these tissues, in which target tissues of insulin become resistant to insulin signaling despite initial compensation by the pancreas. Type 2 DM is the stage of complete or near complete disorder of this relationship, when insulin production no longer fits the organism’s need for glycemic regulation. Each of these target tissues has its own specialized macrophages to maintain important physiological functions, to keep tissue integrity, and, more importantly, the number of macrophages in the tissue undergoes adaptation at each stage of type 2 DM development [14,15]. Tissue macrophages are extremely potent mediators of insulin signaling, sensitivity, and resistance. Macrophages quickly respond to environmental signals and adapt their functions. To date, an important role of macrophage polarization in the development of metabolic diseases has been established [16].

**Figure 1:**
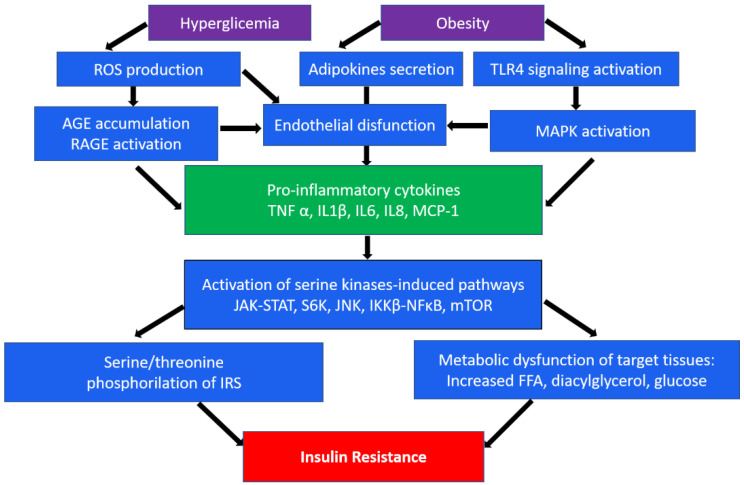
A brief scheme of the main inflammatory mechanisms in the development of insulin resistance. ROS, reactive oxygen species; TLR4, toll-like receptor 4; AGE, advanced glycation end-products; RAGE, AGE receptor; MAPK, mitogen-activated protein (MAP) kinase; TNFα, tumor necrosis factor α; IL, interleukins; MCP-1, monocyte chemoattractant protein-1; JAK-STAT, Janus kinase signal transducers as well as activators of transcriptional signaling pathway; S6k, ribosomal protein S6 kinase; JNK, c-Jun N-terminal kinase signaling pathway; NF-κB, nuclear factor-κB pathway; mTOR, mammalian target of rapamycin; IRS1, insulin receptor substrate 1; FFA, free fatty acids.

An important role in insulin resistance of cells is played by the endoplasmic reticulum (ER). When its homeostasis is disturbed, “ER stress” occurs, which is a key sign of metabolic disorders [17,18]. In obesity, which often accompanies type 2 DM, persistent metabolic pressure leads to disruption of essential ER functions and consequently to impaired cellular health, inflammation, and ultimately metabolic collapse [18]. In addition, hyperglycemia, an independent risk factor for type 2 diabetes, leads to a significant induction of ROS, which causes increased activation of inflammatory pathways [19]. In the occurrence of type 2 diabetes, as well as associated cardiovascular complications, such as atherosclerosis, macrophages/monocytes play a key role as the main regulators of inflammation. Numerous studies show the presence of many levels of regulation of these processes, from cell surface receptors to nuclear receptors, transcription factors, and their coregulators [15,20]. In particular, the mechanisms of impaired insulin action are associated with serine/threonine phosphorylation, which mediates insulin receptor (IR) signaling [21]. In addition, inhibitory phosphorylation can be initiated by pro-inflammatory cytokines (TNFα, IL-1β, and IL-6) secreted by macrophages. These cytokines in turn activate serine kinases such as IκB kinase β (IKKβ), c-Jun N-terminal kinase (JNK), ribosomal protein S6 kinase (S6K), and mammalian target of rapamycin 32 (mTOR32) in adipocytes, which mediate inhibitory phosphorylation of insulin receptor substrate 1 (IRS1), causing insulin resistance [22]. The same kinases play an important role in initiating the immune response through the toll-like receptor (TLR), which, upon activation, secretes the production of various cytokines [23,24]. A brief scheme demonstrating the main inflammatory mechanisms in the development of insulin resistance is shown in Figure 1.

It is known that adipose tissue consists mainly of adipocytes, as well as preadipocytes, lymphocytes, macrophages, fibroblasts, and vascular cells. The content of macrophages is greater in visceral than in subcutaneous adipose tissue, which indicates that the accumulation of visceral fat contributes to insulin resistance and can lead to metabolic diseases. Obesity leads to the change in adipose tissue cellular composition as well as to the activation of immune cells [25]. Adipocyte hypertrophy, hypoxia, and increased cell death due to increased accumulation of lipids, in particular triglycerides, contribute to secretion of pro-inflammatory molecules such as TNF-α, IL-6, IL-8, and MCP-1, adipokines, etc., by adipocytes and immune cells, in particular macrophages, resulting in increased infiltration of circulating monocytes and immune cells into adipose tissue [26,27]. Recruited monocytes differentiate into a pro-inflammatory M1 macrophage phenotype, resulting in an imbalance between M1 and M2 macrophages and reduced anti-inflammatory signals from M2 macrophages. This contributes to greater secretion of pro-inflammatory cytokines and adipokines, and consequently, dysfunction of adipose tissue and decrease in glucose tolerance [28]. Most pro-inflammatory stimuli simultaneously activate the JNK and IKKβ TLR pathways. Activation of JNK and IKKβ/NF-κB can occur due to the influence of pro-inflammatory cytokines such as TNF-α and IL-1β through receptor-mediated mechanisms, as well as non-receptor mechanisms through activation of receptors such as TLR and glycation end products receptor (RAGE) recognition patterns, defined as surface proteins that recognize foreign substances. JNK promotes insulin resistance through phosphorylation of serine residues in IRS-1, and IKKβ induces insulin resistance through transcriptional activation of nuclear factor-κB (NF-κB). Activation of the JNK and NF-κB pathways will lead to the production of pro-inflammatory cytokines and mediators, which further activates these pathways via feed-forward mechanisms [29]. Obesity accompanied by dysfunction of adipose tissue plays a decisive role in the pathogenesis of not only insulin resistance but also liver pathologies such as non-alcoholic steatohepatitis (NASH) and non-alcoholic fatty liver disease (NAFLD) [30]. The relationship between NAFLD and type 2 DM is very complex and bidirectional. On the one hand, NAFLD is an independent risk factor for the development of diabetes. On the other hand, diabetes can contribute to the progression of NASH, NAFLD, liver cirrhosis and, in some cases, hepatocellular carcinoma. In addition, NAFLD is a risk factor for cardiovascular disease, especially in combination with type 2 diabetes [31,32]. The pathogenesis of NAFLD involves the “multiple parallel strikes” hypothesis, which involves insulin resistance, liver triglyceride accumulation, oxidative stress, and processes in adipose tissue that promote a cascade of inflammation and cytokine and adipokine production that leads to liver damage [33].

The major inflammatory pathways playing a key role in diabetes development were demonstrated in mice models. Table 1 shows the major findings of experimental animal studies devoted to the investigation of inflammatory mechanisms in the pathogenesis of type 2 DM.

## 3. Macrophages in Type 2 Diabetes

Current research shows that the study of extracellular, metabolic, and molecular signals associated with macrophage polarization is important in metabolic inflammation and insulin resistance. In obesity, macrophages make up 50% of all adipose tissue cells [40]. Adipose tissue macrophages (ATMs) not only increase in number, but also change their localization, being located around dead adipocytes and forming crown-like structures (CLSs), which exhibit pronounced pro-inflammatory properties [41]. Activation of inflammatory pathways in adipocytes and macrophages is carried out through toll-like receptors, in particular, TLR4. It has been shown that free fatty acids (FFA), which are elevated in obesity, may promote TLR4 signaling, which in turn contributes to obesity-related insulin resistance [42]. In addition, low-density lipoprotein receptors (LDLR) have been shown to be involved in the development of insulin resistance. Increased expression of LDLR in adipocytes of adipose tissue contributes to pro-inflammatory activation and insulin resistance in obesity [43].

Macrophage infiltration into adipose tissue leads to the secretion of pro-inflammatory cytokines such as TNFα, IL-1β, and IL-6, which activate serine kinases in adipocytes, including IKKβ, N-terminal c-Jun kinase (JNK), S6K, and mTOR32. All these kinases trigger inhibitory phosphorylation of IRS1 [22]. Janus kinase (JAK) signal transducers as well as activators of transcriptional (STAT) signaling pathways play an important role in maintaining homeostatic processes. Activation of JAK results in phosphorylation of tyrosine residues in the STAT protein. The JAK-STAT signaling pathway transcriptionally regulates the cytokine signaling suppressor (SOCS), which inhibits JAK and STAT activation and phosphorylation [44].

The general consensus about obesity and type 2 DM is that there is a disbalance in the ratio of M1/M2 macrophages, which leads to an increase in the number of pro-inflammatory M1 macrophages compared to anti-inflammatory M2 macrophages, leading to chronic inflammation and the spread of metabolic dysfunction [14]. Insulin acts on cells through the insulin receptor (IR), located on the surface of insulin-sensitive cells. As a result of IR stimulation, its autophosphorylation occurs, as well as subsequent tyrosine phosphorylation of members of the IRS family, thus initiating signaling events in the cell [45,46]. Dysregulation of insulin signaling, resulting from various factors, is the main mechanism leading ultimately to insulin resistance. In particular, IRS-1 serine phosphorylation leads to increased levels of free fatty acids, diacylglycerol, fatty acyl-CoA, ceramides, and glucose, leading to obesity-associated insulin resistance. Some cytokines, in particular, TNF-α secreted by adipose tissue cells, stimulate the phosphorylation of IRS-1 serine and threonine residues, which reduces IRS-1 tyrosine phosphorylation in response to insulin, as well as the ability of IRS-1 to bind to the insulin receptor, which, in in turn, suppresses signaling [47].

TNF-α and IL-6 increase the expression of SOCS proteins through attenuating insulin signaling by binding to insulin receptors and reducing their ability to phosphorylate IRS proteins. On the other hand, SOCS proteins can directly bind to IRS proteins, resulting in their degradation. In addition, these cytokines can inhibit the expression of IRS-1 at the transcriptional level. Thus, suppression of IRS-1 mRNA expression seems to be the main mechanism involved in altering IRS-1 tyrosine phosphorylation in adipocytes of patients with type 2 DM. IL-1β exerts its pro-inflammatory action by binding to the type I IL-1 receptor and activating the IKK/NF-κB pathway and the three types of mitogen-activated protein (MAP) kinases—extracellular signal-regulated kinase (ERK), JNK, and p38MAPK—which also determines its involvement in insulin resistance [48]. Activation of MAP kinase signaling pathways promotes endothelin-1 (ET-1) secretion, activation of cation pumps, and increased expression of vascular cell adhesion molecule 1 (VCAM-1) and E-selectin. ET-1, in turn, can enhance serine phosphorylation of IRS-1, causing a decrease in PI-3 kinase activity in vascular smooth muscle cells, as well as disrupt insulin-stimulated glucose transporter type 4 (GLUT-4) translocation in adipocytes [49]. Studies have shown that IL-1β levels are elevated in non-diabetic offspring of diabetics and correlate with metabolic syndrome, as well as increased expression of both IL-1β and its receptor in visceral adipose tissue in obese individuals [48].

## 4. The Role of Mitochondria in the Development and Progression of Type 2 Diabetes and Its Vascular Complications

Mitochondria play a key role in metabolic processes in all cells of the body. In endothelial cells, they have a direct effect on the formation of endothelial dysfunction and, therefore, vascular diseases, such as atherosclerosis and diabetic vascular dysfunction, accompanying type 2 diabetes [50,51,52]. The main function of mitochondria is to produce cellular energy using the cyclooxygenase (COX) and associated inner membrane electron transport chain, which produces ATP. In addition, mitochondria are involved in the formation of ROS, calcium and iron homeostasis, steroid biosynthesis, immune cell activation, apoptosis, and inflammation [53,54]. When antioxidant defense mechanisms are disrupted, excessive accumulation of ROS occurs, which not only directly damages cells by oxidizing DNA, proteins, and lipids, but also indirectly damages cells by activating stress-sensitive intracellular signaling pathways, such as NF-κB, p38 MAPK, and JNK/SAPK. Activation of these pathways leads to the development of inflammation and, ultimately, diseases associated with oxidative stress [55,56].

It is known that mitochondria, both in physiological conditions and in pathology, are heterogeneous and have different functional properties, and have differences in morphology, membrane potential, and mitochondrial calcium levels. A pool of mitochondria in an individual cell can represent mitochondria at different stages of development, as well as the consequences of their response to the cellular environment. Mitochondrial heterogeneity is a two-way process in which a lower degree of heterogeneity can be beneficial and can provide the cell with protection and adaptation to biological stress, and a higher degree of heterogeneity can lead to irreversible disease because of the accumulation of dysfunctional or inadequate mitochondria. Studies have described that within the same cell, mitochondria exhibit broad heterogeneity in mitochondrial membrane potential (MMP), which can be generated by the BCL2-associated agonist of cell death (BAD) protein, a member of the proapoptotic BCL-2 family. Glucose-stimulated mitochondrial hyperpolarization has been shown to increase insulin secretion. In addition, mitochondrial heterogeneity provides metabolic flexibility [57].

Under physiological conditions, mitochondria are not static organelles; as a result of continuous cycles of fusion and division, they change their shape and location depending on physiological stimuli. The regulation of mitochondrial dynamics is a complex process that is controlled by several dynamin-related guanosine triphosphate hydrolases (GTPases) that maintain the balance between mitochondrial fusion and fission. Mitofusins MFN1 and MFN2 are responsible for outer mitochondrial membrane (OMM) fusion, while mitochondrial inner membrane (IMM) fusion is regulated by optic atrophy protein 1 (OPA1). Fission proteins include dynamin-related protein 1 (DRP1) and fission protein 1 (FIS1). Mitochondrial fission is essential for the removal of defective mitochondria by mitophagy, which is mediated by (PTEN)-induced putative kinase 1 (PINK1), PARKIN ubiquitin ligase, ubiquitin, and sequestosome-1 (p62/SQSTM1) [58]. It was shown that in patients with type 2 diabetes and obesity, MFN2 expression was reduced, which may be associated with decreased mitochondrial function [59]. Any change in this balance can lead to oxidative stress and mitochondrial dysfunction, including Ca2+ overload, decreased ATP synthesis, and loss of MMP. In turn, this is an important reason for the phenotypic transformation of vascular smooth muscle cells (VSMCs), which can release apoptotic bodies that induce calcification, leading to vessel remodeling and vascular wall stiffness. Ultimately, this can lead to metabolic disorders that underlie the development of type 2 diabetes and its vascular complications [60].

The development of type 2 diabetes, as well as its complications, in particular atherosclerosis, are associated with mtDNA mutations [61,62,63]. Most of the mutations arise because of increased production of ROS near the mitochondrial genome as a result of oxidative stress, which leads to impaired mitochondrial function. In addition, mtDNA mutations are generated by replication errors of mitochondrial DNA polymerase γ and spontaneous base hydrolysis [64]. Recently, a novel m.8561C>G mutation in MT-ATP6/8 (subunits of mitochondrial ATP synthase) has been reported that may be associated with the onset of diabetes mellitus [65]. Another study demonstrated that nuclear-encoded mitochondrial genes (NEMG) have been identified that code for disease-related proteins that act in key mitochondrial pathways. This may confirm the role of genetic variability in the occurrence of mitochondrial dysfunction, not only as a consequence of DM2, but also as its possible cause [66].

## 5. Endothelial Dysfunction in Type 2 Diabetes and Its Vascular Complications

The development of vascular complications in type 2 diabetes is associated with toxic effects caused by hyperglycemia, as well as dyslipidemia caused by obesity, gluco-, and lipotoxicity. Chronic hyperglycemia and dyslipidemia lead to increased production of ROS through the activation of various enzymes such as mitochondrial respiratory chain enzymes, nicotinamide adenine dinucleotide phosphate (NADPH) oxidase (NOX), uncoupled endothelial nitric oxide synthase (eNOS), cyclooxygenase, and xanthine oxidase (XO). ROS-responsive factors increase the production of glyco/lipoxidation end products such as advanced glycation end-product (AGE) and oxidized LDL, which causes endothelial damage and increases intravascular inflammation and leukocyte recruitment, which in turn further increases endothelial dysfunction. ROS production in type 2 DM is increased by accumulation of AGEs and activation of the cellular AGE receptor (RAGE), which promote the secretion of cytokines and stimulate oxidative intermediates under conditions of hyperglycemia [67]. Studies show that AGE/RAGE signaling is involved in diabetes-mediated oxidative stress associated with plaque calcification, endothelial dysfunction, and atherosclerosis progression [68,69]. The damaging effect of ROS is minimized by cellular antioxidant enzymes such as catalase, peroxiredoxins, glutaredoxin (Grx), and glutathione peroxidases (GPx). When these pathophysiological processes are dysregulated, defense mechanisms are reduced, leading to an increase in ROS levels and irreversible damage to key cellular enzymes [70]. Studies show that low antioxidant status predisposes to adverse vascular complications. Thus, a decrease in GPx3 activity is associated with progression of mean carotid intima-media thickness (IMT) and the presence of carotid plaque, which confirms the relationship between GPx3 activity and the pathogenesis of carotid atherosclerosis in patients with type 2 diabetes [71]. It is known that endothelial-cell-derived nitric oxide (NO) plays a protective role in cardiovascular diseases, in particular atherosclerosis, by stimulating vasodilation and inhibiting inflammatory reactions, platelet activation, and aggregation. Violation of the synthesis and/or bioavailability of NO by endothelial cells also leads to endothelial dysfunction. AGEs have been shown to reduce NO production by suppressing the expression of endothelial NO synthase. In addition, oxidative stress caused by AGE-RAGE can also inactivate NO and lead to increased production of peroxynitrite, a toxic by-product of NO. AGE-RAGE stimulates the formation of the endogenous NO synthase inhibitor asymmetric dimethylarginine (ADMA), which also causes endothelial dysfunction [72].

**Figure 2:**
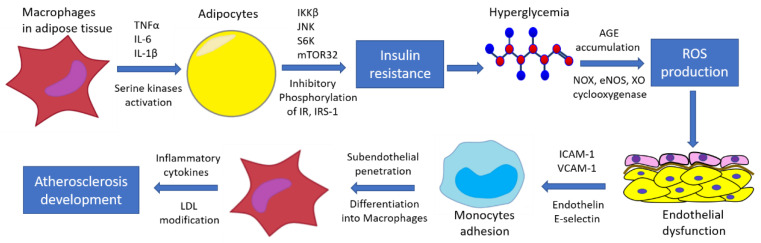
Mechanisms of atherosclerosis development in diabetes.

Hyperglycemia and dyslipidemia also promote monocyte adhesion to endothelial cells by inhibiting nitric oxide production and increasing levels of endothelin-1, E-selectin, intercellular adhesion molecule 1 (ICAM-1) and VCAM-1, ROS, angiotensin II, and a plasminogen activator inhibitor [73]. Further, monocytes penetrate into the subendothelial space, differentiating into macrophages that secrete pro-inflammatory cytokines [74]. As a result, LDLs are converted into modified atherogenic LDLs, and oxidation probably occurs in the last stages of modification. These modified LDLs are then taken up by macrophages, leading to foam cell formation and atherogenesis. It is known that AGEs affect the activation of the endothelium and the expression of adhesion molecules, promoting the penetration of monocytes into the subendothelial space, and also enhancing the release of cytokines by macrophages, thereby maintaining the pro-inflammatory effect [75,76]. Glycation of LDL can be considered as a type of the atherogenic LDL modification [77]. Figure 2 demonstrates the pathogenesis of atherosclerosis development in a diabetes condition that shows the involvement of inflammatory mechanisms at different stages of the process.

It is known that AGEs are formed during periods of hyperglycemia, are poorly metabolized, and slowly accumulate over many years with inadequate glucose control in DM. This is the so-called “metabolic memory”, defined as the long-term influence of the initial glycemic status on the development of diabetic vascular complications, which can accelerate the progression of vascular complications in patients with diabetes mellitus [78].

In addition to being involved in the formation of atherosclerotic lesions, endothelial dysfunction is associated with the development of other diabetic cardiovascular complications of non-ischemic origin. Endothelial-derived cardio-active factors, such as NO, endothelin-1, neuregulin-1 (NRG-1), angiotensin II, prostaglandins, and others, regulate cardiomyocyte activity [79]. In diabetic conditions, endothelial dysfunction leads to disturbed cardiomyocyte metabolism and microvascular coronary disorder, which are the major mechanisms of diabetic cardiomyopathy [80]. The deposition of microvascular AGEs in myocardium causes vascular inflammation and inhibits NO production, ROS production in cardiomyocytes by NADPH oxidase, increased connective tissue crosslinking, and fibrosis development, resulting in predisposition to left ventricular remodeling and diastolic dysfunction [80,81]. Recent meta-analysis demonstrates the association of myocardial fibrosis with DM in clinical studies due to impaired glycemic control, but further investigation of this relationship mechanisms is needed [82]. In addition, endothelial dysfunction in conjunction with increased activation of the renin–angiotensin–aldosterone system and immune dysregulation underlie the pathophysiology of arterial hypertension, one of the major comorbidities of diabetes [83]. Thus, diabetic mechanisms cause heart failure development due to the direct role in cardiac metabolism dysregulation and indirectly through arterial hypertension and coronary atherosclerosis [84].

## 6. Current Therapeutic Strategies for the Treatment of Type 2 Diabetes and Its Vascular Complications

Current traditional classes of drugs for the treatment of type 2 diabetes include sulfonylurea preparations (enhance the release of insulin from the pancreatic islets); biguanides (reduce glucose production by the liver); agonists of the peroxisome proliferation-activated receptor (PPAR) (enhance the action of insulin); α-glucosidase inhibitors (prevent the absorption of glucose in the intestine); and sodium–glucose cotransporter inhibitors (SGLT2). These classes of drugs are prescribed either as monotherapy or in combination with other hypoglycemic agents [85,86].

### 6.1. Biguanides

The drug of first choice in the treatment of type 2 diabetes is biguanides (the classic representative of metformin), the action of which is due to the inhibition of gluconeogenesis in the liver, which leads to a decrease in glucose production, as well as improved insulin signaling and a subsequent increase in glucose uptake by skeletal myocytes. Biguanides do not increase the risk of hypoglycemia and weight gain and may also reduce the development and progression of certain types of cardiovascular disease [87,88]. However, they have undesirable gastrointestinal side effects including diarrhea, nausea, vomiting, and discomfort, and may also cause lactic acidosis and malabsorption of vitamin B12 [89]. “Classic” sulfonylurea drugs have a high risk of hypoglycemia and are associated with weight gain. Because T2DM is a progressive disease, most patients require additional therapy [90].

### 6.2. Alpha-Glucosidase Inhibitors

Alpha-glucosidase inhibitors (acarbose, miglitol, and voglibose) have been proposed as treatments for type 2 diabetes, obesity, and atherosclerosis [91]. They reduce the hydrolytic breakdown of the non-reducing ends of dietary oligosaccharides and reduce the release of α-glucose, which leads to slower carbohydrate digestion. As a result, glucose absorption in the small intestine decreases, which plays an important role in the control of postprandial hyperglycemia (PPG) [92,93]. A number of studies have shown that alpha-glucosidase inhibitors reduce the concentration of gastric inhibitory polypeptide (GIP), which leads to a decrease in insulin resistance and a decrease in obesity [94]. Since α-glucosidase inhibitors most effectively reduce PPG, which makes a significant contribution to the development of cardiovascular complications of DM, drugs in this group can play an important role both as first-line therapy and second or third line [95].

### 6.3. GLP-1 Agonists

Currently, glucagon-like peptide-1 (GLP-1) agonists represent a well-established class of hypoglycemic agents that have great potential for further development in the treatment of type 2 diabetes. GLP-1 belongs to the family of incretin hormones, and it is able to increase insulin secretion in a glucose-dependent manner and suppress glucagon secretion during periods of hyperglycemia. GLP-1 agonists are recommended as the preferred first injectable hypoglycemic therapy for type 2 diabetes prior to insulin treatment because they are able to lower glucose levels comparable to insulin, but with a lower risk of hypoglycemia [96]. An additional advantage of this group of drugs is that they provide a significant improvement in HbA1c levels, have a low risk of hypoglycemia, improve satiety and promote weight loss due to delayed gastric emptying, and have a central effect on the satiety center [97]. Structural differences among GLP-1 agonists affect their duration of action, while composition and dosage affect hypoglycemic efficacy, weight loss, and side effect profile [98]. Various chemical modifications of GLP-1 explain individual pharmacokinetic properties of different GLP-1RAs, which are classified into short-acting and long-acting formulations. A feature of short-acting drugs is the postprandial decrease in blood glucose levels resulting from delayed gastric emptying, which leads to a decrease in the rate of glucose entry into the duodenum and, subsequently, in blood. In addition, short-acting GLP-1RAs have been shown to reduce postprandial blood lipid levels. Long-acting GLP agonists promote better glycemic control than short-acting agonists by maintaining higher fasting insulin levels. In addition, high levels of long-acting GLP-1 receptor agonists in plasma lead to a greater decrease in HbA1c levels. Unlike short range agonists, long-acting GLP-1RA do not have a significant effect on gastric motility with long-term administration, which is probably associated with tachyphylaxis, i.e., the effect of these compounds on gastric emptying decreases rapidly over time as a result of continuous activation of the GLP-1 receptor. The choice of using short-acting or long-acting agonists depends on the disease profile and individual characteristics of the patient [99]. The effectiveness of GLP-1 receptor agonists, as well as their side effect profiles, likely depend on the frequency of their administration [100]. The most frequent side effects are effects observed in the gastrointestinal tract (nausea, vomiting, diarrhea); local reactions at the injection site are possible, since they are administered subcutaneously, as well as hypoglycemia [101]. GLP-1 receptor agonists have beneficial effects on known cardiovascular risk factors such as body weight, glycemic control, fasting, and postprandial lipoprotein levels, and may reduce mild inflammation and improve plaque stability [102]. The anti-atherosclerotic effect of GLP-1 has been shown in experiments on animals and cell cultures. Abundant expression of GLP-1 in endothelial cells, monocytes, and macrophages, which play an important role in the development of atherosclerosis, may cause effects that potentially prevent the formation of atherosclerotic plaques, which requires further study [103]. GLP-1RA has been shown to reduce ROS production, monocyte- and macrophage-mediated oxLDL activation, and subsequent activation of adhesion molecules such as VCAM-1, MCP-1, E-selectin, and ICAM-1, which leads to a decrease in monocyte accumulation in the vascular wall, which ultimately leads to slower progression and stabilization of plaques [96].

An important property of GLP-1 receptor agonists is their resistance to degradation and inhibition by the enzyme dipeptidyl peptidase-4 (DPP-4). This is of great importance for the development of new GLP-1RAs, and also leads to the development of a new class of drugs—DPP-4 inhibitors. DPP-4 inhibitors are a group of oral medications that use a physiological mechanism to achieve glycemic control by stimulating insulin secretion and decreasing glucagon secretion during the acute phase, as well as improving long-term B-cell function and regeneration [104,105,106]. They are well tolerated by patients and have moderate hypoglycemic efficacy as well as minimal risk of side effects such as hypoglycemia and weight gain [107]. All DPP-4 inhibitors reduce the degradation of GLP-1 and therefore are associated with increased endogenous concentrations of human GLP-1, which may have similar physiological effects to GLP-1RA [108]. This group of drugs is of great interest for further study. They can help treat patients who cannot achieve adequate control with conventional therapy for type 2 diabetes and can be used in individuals at risk to prevent the development of diabetes [104].

One of the newest and most promising classes of drugs for the treatment of type 2 diabetes are glucose-dependent insulinotropic polypeptide (GIP) receptor agonists, which, like GLP-1, belong to incretin hormones. GIPR agonists increase whole-body insulin sensitivity and also improve adipose tissue health associated with increased insulin signaling by adipocytes, reduced infiltration of pro-inflammatory immune cells, and decreased lipid accumulation and oxidation [109]. The development of therapeutic agents for the treatment of type 2 diabetes with simultaneous action on the GIP and GLP-1 receptors, that is, dual agonists of the GIP/GLP-1 receptors, looks promising [110]. The dual agonist of the GIP/GLP-1 receptor Tirzepatide has been shown in some studies to improve glycemic control, weight loss, and decrease appetite in patients [111]. It is important that GIP agonists exhibit some anti-atherosclerotic activity due to a decrease in oxidative stress and release of inflammatory cytokines [112].

### 6.4. PPAR Agonists

The antidiabetic drugs thiazolidinediones, PPAR agonists known as “glitazones”, have been proven well [113]. PPARs are a family of ligand-activated nuclear hormone receptors (NRs) that act as ligand-inducible transcription factors. There are several types of PPAR receptors that have different effects on target cells: PPARα affects the metabolism of fatty acids, reducing lipid levels; PPARδ (also referred to as PPARβ) is involved in fatty acid oxidation and also regulates blood glucose and cholesterol levels; PPARγ is involved in the regulation of adipogenesis, lipid biosynthesis, energy balance, inflammatory processes, and is also associated with cell cycle regulation and the development of insulin sensitivity [114,115]. PPARs are expressed in various tissues, as PPARα is highly expressed in hepatocytes, enterocytes, monocytes/macrophages, and endothelial cells; PPARβ/δ is in skeletal muscles, adipocytes, macrophages, lungs, brain and skin; PPARγ (PPARγ-1, γ-2, and γ-3) are secreted in skeletal muscle, liver, heart, and intestines, with PPARγ1 expressed in a wide range of tissues, PPARγ2 in adipose tissue, and PPARγ3 in macrophages, colon, and white adipose tissue (WAT) [116]. Natural ligands for PPAR receptors are fatty acids (FA) and eicosanoids [117]. Thiazolidinediones directly activate PPARγ receptors, which facilitates the differentiation of mesenchymal stem cells into adipocytes, stimulates lipogenesis in peripheral adipocytes, reduces triglyceride levels in the liver and peripheral tissues, reduces the activity of visceral adipocytes, and increases the level of adiponectin [118]. This contributes to a decrease in insulin resistance, as well as such important effects as a decrease in dyslipidemia, anti-inflammatory activity, as well as an improvement in endothelial function [118,119]. It was found that PPARγ agonists suppress the M1 phenotype by inhibiting the expression of pro-inflammatory cytokines, tumor necrosis factor-α, and interleukin (IL)-1β and IL-6, and that M2 differentiation of macrophages leads to an increase in PPARγ expression, which is one of the mechanisms of their anti-inflammatory and anti-atherosclerotic activity [120]. In addition, it has been shown that PPARγ regulates the activity of MAPK, in particular, reduces the activity of N-terminal c-Jun kinase (JNK) MAPK and p38 in the colon, which leads to suppression of the expression of pro-inflammatory genes [121]. Another study demonstrates a positive effect of PPAR activation on the nuclear transcription factor NF-κB, which mediates many inflammatory processes [122]. The positive effect of thiazolidinediones on endothelial function is manifested by an increase in insulin-dependent release of endothelial nitric oxide, an increase in the expression of vascular endothelial growth factor, and a decrease in the expression of endothelin-1 [123].

Thiazolidinediones are quite well tolerated by patients, since they do not cause significant side effects in the gastrointestinal tract, unlike other hypoglycemic agents, but still have a number of side effects, such as weight gain and fluid retention, which can lead to peripheral edema and heart failure [124]. The most well-known representatives of the thiazolidinedione used in clinical practice, rosiglitazone and pioglitazone, are PPARγ agonists. Troglitazone is the first of this class to have fatal hepatotoxicity and is therefore not currently used. Dual, selective, or triple agonists for PPARα/γ/δ are currently being considered, as PPARα and PPARδ have important fat-burning activity and can overcome the side effects of PPARγ agonists [125,126]. Studies using surrogate markers of atherosclerosis such as carotid IMT show a reduction in the progression of carotid IMT in individuals treated with thiazolidinediones [127,128].

### 6.5. SGLT-2 Inhibitors

In recent years, the use of SGLT-2 inhibitors has increased and is considered as a second-line agent after classical metformin for the treatment of patients with type 2 diabetes with established cardiovascular disease. These drugs have a positive effect on the glycemic profile and cardiovascular status in diabetes; in addition, they have metabolic benefits (weight loss), lower blood pressure, and improve kidney function [129,130]. The main representatives of this group of drugs used in clinical practice for the treatment of type 2 diabetes are canagliflozin, dapagliflozin, empagliflozin, ertugliflozin, and sotagliflozin [131,132]. SGLT2s have an insulin-independent mechanism for lowering blood glucose levels, and they promote the excretion of glucose in the urine by inhibiting the reabsorption of glucose from the urine in the proximal tubules of the kidneys [133,134,135]. In addition, SGLT2 lowers blood pressure. The mechanism of this action is not entirely clear, but probably depends on several factors, including weight loss, the diuretic effect resulting from increased sodium excretion during SGLT2 blockade, and improvement in arterial stiffness [136,137,138]. Studies have shown a direct cardioprotective effect of this group of drugs on the myocardium. In addition, SGLT2 inhibitors have an anti-inflammatory vascular effect, accompanied by a decrease in the expression of inflammatory molecules, such as monocyte chemoattractant protein-1, vascular cell adhesion molecules-1, and intercellular adhesion molecule [139]. In addition, SGLT2 inhibitors have been noted to reduce glucose-induced high levels of oxidative stress and RAGE induction [140].

It should be noted that the main groups of modern antidiabetic preparations have various anti-inflammatory effects, which can largely determine their effectiveness in the treatment of diabetes and associated comorbidities [141]. The major anti-inflammatory mechanisms of action of antidiabetic preparations are presented in Table 2.

## 7. Conclusions

This review highlights currently published data on the inflammatory mechanisms underlying the pathogenesis of type 2 diabetes mellitus and associated vascular complications. Due to the multifactorial nature of the development of these pathologies, it is impossible to single out the main pathogenetic mechanism leading to their development. Studies show macrophage infiltration of adipose tissue and production of pro-inflammatory molecules in obesity triggering a cascade of inflammatory responses, as well as the direct influence of hyperglycemia and hyperlipidemia on activation of immune cells and inflammatory pathways. These factors lead to the development of metabolic dysfunction and insulin resistance of target tissues that is a key step in the pathogenesis of type 2 DM. Data on the mechanisms of the development of type 2 diabetes and its vascular complications, in particular atherosclerosis, open up broad prospects for the further development of new diagnostic and therapeutic approaches. Modern antidiabetic preparations possess anti-inflammatory effects which allow ameliorating cardiovascular risk, but future investigation of their cardioprotective action in diabetic patients is still required.

## Figures and Tables

**Table 1:** Some key inflammatory pathways of diabetes proven in mice.

Pathway	Model	Findings
NOD-, LRR- and pyrin-domain-containing protein 3 (NLRP3) inflammasome activation: protein complex triggering inflammatory mediator production	Streptozotocin-induced diabetic mice	Microglia NLRP3 proteins were highly expressed, and serum cytokines IL-1β, IL6, IL18, and TNFα were increased in streptozotocin-induced diabetic mice [34]
Endothelial NF-κB signaling: complex of transcription factors that regulate cytokine gene expression and the inflammatory response	Diabetic C57BL/KsJ db/db mice compared to healthy mice	The mRNA and protein levels of NF-κB and TLR4 were significantly higher in the db/db mice compared to normal control group [35]
	E-DNIκB mice (transgenic mice expressing dominant-negative IκB under the Tie2 promoter/enhancer)	Endothelial NF-κB inhibition ameliorates insulin resistance and improves glucose homeostasis via reduced aortic expression of adhesion molecules, upregulation of eNOS signaling, reduced macrophage infiltration, and iNOS expression in adipose tissue [36]
Major inflammatory cytokines secretion: IL-1β, IL-6, IL-18, and TNF-α	Diabetic C57BL/KsJ db/db mice compared to healthy mice	The serum levels of IL-1β, IL-6, IL-18, and TNF-α in the db/db mice were significantly higher compared to healthy control group [35]
	Male db/db mice	IL-1β, IL-18, and TNF-α levels were increased in liver of db/db mice compared to healthy mice [37]
JNK pathway activation: one of the major signaling cassettes of the mitogen-activated protein kinase (MAPK) signaling pathway	Apolipoprotein E/low-density lipoprotein receptor double-knockout (AL) mice	Hepatic inflammation and dyslipidemia were increased in AL mice on 35-week Western diet (WD) compared with wild-type mice on WD through activation of NF-κB, Stat3, JNK signaling pathways [38]
Chaperone-mediated autophagy (CMA): catabolic pathway for selective degradation of cytosolic proteins in lysosomes	Knockout mice 4–6 months old with selectively blocked CMA in liver	Key enzymes in carbohydrate and lipid metabolism are normally degraded by CMA while CMA block leads to peripheral adiposity, increased energy expenditure, and altered glucose homeostasis [39]

**Table 2:** Metabolic and anti-inflammatory effects of modern antidiabetic preparations.

Group of Preparation	Mechanism of Action	Metabolic Effects	Anti-Inflammatory Effects
Sulfonylurea preparations	Bind to the sulfonylurea receptor (SUR) of ATP-sensitive potassium channel on pancreatic β cells	Enhance the release of insulin from the pancreatic islets	- Inhibit the NLRP3 inflammasome [142], decrease production of pro-inflammatory cytokines (IL-1β, IL-6, and TNF-α) [143]; - Inhibit AGEs-induced pro-inflammatory mediators (NO, reactive oxygen species, i-NOS) [143]; - Enhance production of anti-inflammatory cytokines (IL-10 and TGF-β) [143].
Biguanides	Block the breakdown of fatty acids through activation of AMP-dependent protein kinase	Reduce glucose production in liver by decreasing gluconeogenesis and stimulating glycolysis	- Activation of AMP-activated protein kinase (AMPK) [144,145]; - Inhibit mTOR and NF-κB pro-inflammatory signaling [145]; - Reduce inflammatory cytokines IL-6 and TNF-α [146].
PPAR agonists	Activate PPARα/γ/δ receptors	Enhance insulin effects, decrease insulin resistance, decrease dyslipidemia	- Downregulate the inflammatory pathway NF-κB [147]; - Regulate adipokine production and secretion [148]; - Inhibit of pro-inflammatory molecules in liver [149].
α-Glucosidase inhibitors	Inhibit enzymes in the small intestine	Prevent the absorption of glucose in the intestine	- Decrease TNF-α and other inflammatory mediators [150]; - Ameliorate vascular endothelial dysfunction [151]; - Decrease C-reactive protein (CRP) [151].
SGLT2 inhibitors	inhibit SGLT-2	Promote the excretion of glucose in the urine by inhibiting the reabsorption of glucose from the urine in the proximal tubules of the kidneys	- Improve endothelial function [12]; - Reduce inflammatory mediators IL-6, TNF-α, MCP-1, and CRP in plasma and liver [152]; - Inhibit NLRP3 inflammasome [153]; - Cause M2 macrophage polarization [153].
GLP-1 agonists (GLP-1RA)	Activate GLP-1 receptor	Increase insulin secretion in a glucose-dependent manner and suppress glucagon secretion	- Reduce production of IL-6, TNF-α, and MCP-1 in adipose tissue [154]; - Inhibit NF-κB and JNK pathways [155]; - Decrease CRP [154].
DPP-4 inhibitors	Inhibit DPP-4 receptor	Stimulate insulin secretion and decrease glucagon secretion, improve B-cell function and regeneration	- Reduce inflammatory cytokines IL-2, TNF-α, IL-1β, and IL-6 gene expression [156]; - Decrease NLRP3 inflammasome and TLR-4 activity [157]; - Suppress NF-κB activation [158].

## Data Availability

Not applicable.

## References

[1] IDF Diabetes Atlas Tenth Edition. https://diabetesatlas.org/.

[2] Bloomgarden Z. (2020). Does Glycemic Control Affect Outcome of COVID-19?. J. Diabetes.

[3] Barron E., Bakhai C., Kar P., Weaver A., Bradley D., Ismail H., Knighton P., Holman N., Khunti K., Sattar N. (2020). Associations of Type 1 and Type 2 Diabetes with COVID-19-Related Mortality in England: A Whole-Population Study. Lancet Diabetes Endocrinol..

[4] Rana J.S., Dunning A., Achenbach S., Al-Mallah M., Budoff M.J., Cademartiri F., Callister T.Q., Chang H.J., Cheng V.Y., Chinnaiyan K. (2012). Differences in Prevalence, Extent, Severity, and Prognosis of Coronary Artery Disease among Patients with and without Diabetes Undergoing Coronary Computed Tomography Angiography: Results from 10,110 Individuals from the CONFIRM (COronary CT Angiography Evaluation for Clinical Outcomes): An InteRnational Multicenter Registry. Diabetes Care.

[5] Paneni F., Beckman J.A., Creager M.A., Cosentino F. (2013). Diabetes and Vascular Disease: Pathophysiology, Clinical Consequences, and Medical Therapy: Part I. Eur. Heart J..

[6] Selvin E., Marinopoulos S., Berkenblit G., Rami T., Brancati F.L., Powe N.R., Golden S.H. (2004). Meta-Analysis: Glycosylated Hemoglobin and Cardiovascular Disease in Diabetes Mellitus. Ann. Intern. Med..

[7] Katakami N. (2018). Mechanism of Development of Atherosclerosis and Cardiovascular Disease in Diabetes Mellitus. J. Atheroscler. Thromb..

[8] Santilli F., D’Ardes D., Davì G. (2015). Oxidative Stress in Chronic Vascular Disease: From Prediction to Prevention. Vasc. Pharm..

[9] Williamson R.T. (1901). On the Treatment of Glycosuria and Diabetes Mellitus with Sodium Salicylate. Br. Med. J..

[10] Reid J., Macdougall A.I., Andrews M.M. (1957). Aspirin and Diabetes Mellitus. Br. Med. J..

[11] Shimobayashi M., Albert V., Woelnerhanssen B., Frei I.C., Weissenberger D., Meyer-Gerspach A.C., Clement N., Moes S., Colombi M., Meier J.A. (2018). Insulin Resistance Causes Inflammation in Adipose Tissue. J. Clin. Investig..

[12] Johnson A.M.F., Olefsky J.M. (2013). The Origins and Drivers of Insulin Resistance. Cell.

[13] Wu H., Ballantyne C.M. (2017). Skeletal Muscle Inflammation and Insulin Resistance in Obesity. J. Clin. Investig..

[14] Castoldi A., de Souza C.N., Saraiva Câmara N.O., Moraes-Vieira P.M. (2016). The Macrophage Switch in Obesity Development. Front. Immunol..

[15] Drareni K., Gautier J.F., Venteclef N., Alzaid F. (2019). Transcriptional Control of Macrophage Polarisation in Type 2 Diabetes. Semin. Immunopathol..

[16] Martinez F.O., Gordon S. (2014). The M1 and M2 Paradigm of Macrophage Activation: Time for Reassessment. F1000Prime Rep..

[17] Ozawa K., Miyazaki M., Matsuhisa M., Takano K., Nakatani Y., Hatazaki M., Tamatani T., Yamagata K., Miyagawa J.I., Kitao Y. (2005). The Endoplasmic Reticulum Chaperone Improves Insulin Resistance in Type 2 Diabetes. Diabetes.

[18] Lemmer I.L., Willemsen N., Hilal N., Bartelt A. (2021). A Guide to Understanding Endoplasmic Reticulum Stress in Metabolic Disorders. Mol. Metab..

[19] Lin Y., Berg A.H., Iyengar P., Lam T.K.T., Giacca A., Combs T.P., Rajala M.W., Du X., Rollman B., Li W. (2005). The Hyperglycemia-Induced Inflammatory Response in Adipocytes: The Role of Reactive Oxygen Species. J. Biol. Chem..

[20] Kirichenko T.V., Markina Y.V., Sukhorukov V.N., Khotina V.A., Wu W.K., Orekhov A.N. (2020). A Novel Insight at Atherogenesis: The Role of Microbiome. Front. Cell Dev. Biol..

[21] Tanti J.F., Grémeaux T., van Obberghen E., le Marchand-Brustel Y. (1994). Serine/Threonine Phosphorylation of Insulin Receptor Substrate 1 Modulates Insulin Receptor Signaling. J. Biol. Chem..

[22] Haeusler R.A., McGraw T.E., Accili D. (2018). Biochemical and Cellular Properties of Insulin Receptor Signalling. Nat. Rev. Mol. Cell Biol..

[23] Takeda K., Akira S. (2005). Toll-like Receptors in Innate Immunity. Int. Immunol..

[24] Yaglova N.V., Obernikhin S.S., Yaglov V.V., Nazimova S.V. (2021). Role of Skin Dendritic and Mast Cells Communications in Triggering Immune Reactions. Clin. Exp. Morphol..

[25] Ouchi N., Parker J.L., Lugus J.J., Walsh K. (2011). Adipokines in Inflammation and Metabolic Disease. Nat. Rev. Immunol..

[26] Taylor E.B. (2021). The Complex Role of Adipokines in Obesity, Inflammation, and Autoimmunity. Clin. Sci..

[27] Leal V.d.O., Mafra D. (2013). Adipokines in Obesity. Clin. Chim. Acta.

[28] Kraakman M.J., Murphy A.J., Jandeleit-Dahm K., Kammoun H.L. (2014). Macrophage Polarization in Obesity and Type 2 Diabetes: Weighing down Our Understanding of Macrophage Function?. Front. Immunol..

[29] Shoelson S.E., Lee J., Goldfine A.B. (2006). Inflammation and Insulin Resistance. J. Clin. Investig..

[30] Herck M.A.V., Weyler J., Kwanten W.J., Dirinck E.L., Winter B.Y.D., Francque S.M., Vonghia L. (2019). The Differential Roles of T Cells in Non-Alcoholic Fatty Liver Disease and Obesity. Front. Immunol..

[31] Vinué Á., Herrero-Cervera A., González-Navarro H. (2018). Understanding the Impact of Dietary Cholesterol on Chronic Metabolic Diseases through Studies in Rodent Models. Nutrients.

[32] Anstee Q.M., Targher G., Day C.P. (2013). Progression of NAFLD to Diabetes Mellitus, Cardiovascular Disease or Cirrhosis. Nat. Rev. Gastroenterol. Hepatol..

[33] Vonghia L., van Herck M.A., Weyler J., Francque S. (2019). Targeting Myeloid-Derived Cells: New Frontiers in the Treatment of Non-Alcoholic and Alcoholic Liver Disease. Front. Immunol..

[34] Li Y., Zhang H., Liu M., Guo W., Yu L. (2021). Microglia NLRP3 Inflammasomes Activation Involving Diabetic Neuroinflammation in Diabetic Mice and BV2 Cells. Curr. Pharm. Des..

[35] Zhao W., Deng C., Han Q., Xu H., Chen Y. (2020). Carvacrol May Alleviate Vascular Inflammation in Diabetic Db/Db Mice. Int. J. Mol. Med..

[36] Hasegawa Y., Saito T., Ogihara T., Ishigaki Y., Yamada T., Imai J., Uno K., Gao J., Kaneko K., Shimosawa T. (2012). Blockade of the Nuclear Factor-ΚB Pathway in the Endothelium Prevents Insulin Resistance and Prolongs Life Spans. Circulation.

[37] Wang A., Gong Y., Pei Z., Jiang L., Xia L., Wu Y. (2022). Paeoniflorin Ameliorates Diabetic Liver Injury by Targeting the TXNIP-Mediated NLRP3 Inflammasome in Db/Db Mice. Int. Immunopharmacol..

[38] Kampschulte M., Stöckl C., Langheinrich A.C., Althöhn U., Bohle R.M., Krombach G.A., Stieger P., Churin Y., Kremer S., Dierkes C. (2014). Western Diet in ApoE-LDLR Double-Deficient Mouse Model of Atherosclerosis Leads to Hepatic Steatosis, Fibrosis, and Tumorigenesis. Lab. Investig..

[39] Schneider J.L., Suh Y., Cuervo A.M. (2014). Deficient Chaperone-Mediated Autophagy in Liver Leads to Metabolic Dysregulation. Cell Metab..

[40] Weisberg S.P., McCann D., Desai M., Rosenbaum M., Leibel R.L., Ferrante A.W. (2003). Obesity Is Associated with Macrophage Accumulation in Adipose Tissue. J. Clin. Investig..

[41] Boutens L., Stienstra R. (2016). Adipose Tissue Macrophages: Going off Track during Obesity. Diabetologia.

[42] Shi H., Kokoeva M.V., Inouye K., Tzameli I., Yin H., Flier J.S. (2006). TLR4 Links Innate Immunity and Fatty Acid-Induced Insulin Resistance. J. Clin. Investig..

[43] Shin K.C., Hwang I., Choe S.S., Park J., Ji Y., Kim J.I., Lee G.Y., Choi S.H., Ching J., Kovalik J.P. (2017). Macrophage VLDLR Mediates Obesity-Induced Insulin Resistance with Adipose Tissue Inflammation. Nat. Commun..

[44] Wunderlich C.M., Hövelmeyer N., Wunderlich F.T. (2013). Mechanisms of Chronic JAK-STAT3-SOCS3 Signaling in Obesity. JAKSTAT.

[45] Hotamisligil G.S., Peraldi P., Budavari A., Ellis R., White M.F., Spiegelman B.M. (1996). IRS-1-Mediated Inhibition of Insulin Receptor Tyrosine Kinase Activity in TNF-Alpha- and Obesity-Induced Insulin Resistance. Science.

[46] Saltiel A.R., Pessin J.E. (2002). Insulin Signaling Pathways in Time and Space. Trends Cell Biol..

[47] Aguirre V., Werner E.D., Giraud J., Lee Y.H., Shoelson S.E., White M.F. (2002). Phosphorylation of Ser307 in Insulin Receptor Substrate-1 Blocks Interactions with the Insulin Receptor and Inhibits Insulin Action. J. Biol. Chem..

[48] Jager J., Grémeaux T., Cormont M., le Marchand-Brustel Y., Tanti J.F. (2007). Interleukin-1beta-Induced Insulin Resistance in Adipocytes through down-Regulation of Insulin Receptor Substrate-1 Expression. Endocrinology.

[49] Simsek S., van den Oever I.A.M., Raterman H.G., Nurmohamed M.T. (2010). Endothelial Dysfunction, Inflammation, and Apoptosis in Diabetes Mellitus. Mediat. Inflamm..

[50] Pangare M., Makino A. (2012). Mitochondrial Function in Vascular Endothelial Cell in Diabetes. J. Smooth Muscle Res..

[51] Tang X., Luo Y.X., Chen H.Z., Liu D.P. (2014). Mitochondria, Endothelial Cell Function, and Vascular Diseases. Front. Physiol..

[52] Salnikova D., Orekhova V., Grechko A., Starodubova A., Bezsonov E., Popkova T., Orekhov A. (2021). Mitochondrial Dysfunction in Vascular Wall Cells and Its Role in Atherosclerosis. Int. J. Mol. Sci..

[53] Suárez-Rivero J.M., Pastor-Maldonado C.J., Povea-Cabello S., Álvarez-Córdoba M., Villalón-García I., Talaverón-Rey M., Suárez-Carrillo A., Munuera-Cabeza M., Sánchez-Alcázar J.A. (2021). From Mitochondria to Atherosclerosis: The Inflammation Path. Biomedicines.

[54] Markin A.M., Khotina V.A., Zabudskaya X.G., Bogatyreva A.I., Starodubova A.V., Ivanova E., Nikiforov N.G., Orekhov A.N. (2021). Disturbance of Mitochondrial Dynamics and Mitochondrial Therapies in Atherosclerosis. Life.

[55] Newsholme P., Haber E.P., Hirabara S.M., Rebelato E.L.O., Procopio J., Morgan D., Oliveira-Emilio H.C., Carpinelli A.R., Curi R. (2007). Diabetes Associated Cell Stress and Dysfunction: Role of Mitochondrial and Non-Mitochondrial ROS Production and Activity. J. Physiol..

[56] Newsholme P., Gaudel C., Krause M. (2012). Mitochondria and Diabetes. An Intriguing Pathogenetic Role. Adv. Exp. Med. Biol..

[57] Ngo J., Osto C., Villalobos F., Shirihai O.S. (2021). Mitochondrial Heterogeneity in Metabolic Diseases. Biology.

[58] Rovira-Llopis S., Bañuls C., Diaz-Morales N., Hernandez-Mijares A., Rocha M., Victor V.M. (2017). Mitochondrial Dynamics in Type 2 Diabetes: Pathophysiological Implications. Redox Biol..

[59] Wada J., Nakatsuka A. (2016). Mitochondrial Dynamics and Mitochondrial Dysfunction in Diabetes. Acta Med. Okayama.

[60] Li M., Zhu Y., Jaiswal S.K., Liu N.F. (2021). Mitochondria Homeostasis and Vascular Medial Calcification. Calcif. Tissue Int..

[61] Permana Maksum I., Saputra S.R., Indrayati N., Yusuf M., Subroto T. (2017). Bioinformatics Study of m.9053G>A Mutation at the ATP6 Gene in Relation to Type 2 Diabetes Mellitus and Cataract Diseases. Bioinform. Biol. Insights.

[62] Kirichenko T.V., Ragino Y.I., Voevoda M.I., Urazalina S.J., Khasanova Z.B., Orekhova V.A., Sinyov V.V., Sazonova M.A., Orekhov A.N., Sobenin I.A. (2020). Data on Association of Mitochondrial Heteroplasmy with Carotid Intima-Media Thickness in Subjects from Russian and Kazakh Populations. Data Brief.

[63] Kirichenko T.V., Ryzhkova A.I., Sinyov V.V., Sazonova M.D., Orekhova V.A., Karagodin V.P., Gerasimova E.V., Voevoda M.I., Orekhov A.N., Ragino Y.I. (2020). Impact of Mitochondrial DNA Mutations on Carotid Intima-Media Thickness in the Novosibirsk Region. Life.

[64] Markin A.M., Sobenin I.A., Grechko A.V., Zhang D., Orekhov A.N. (2020). Cellular Mechanisms of Human Atherogenesis: Focus on Chronification of Inflammation and Mitochondrial Mutations. Front. Pharm..

[65] Kytövuori L., Lipponen J., Rusanen H., Komulainen T., Martikainen M.H., Majamaa K. (2016). A Novel Mutation m.8561C>G in MT-ATP6/8 Causing a Mitochondrial Syndrome with Ataxia, Peripheral Neuropathy, Diabetes Mellitus, and Hypergonadotropic Hypogonadism. J. Neurol..

[66] Maude H., Lau W., Maniatis N., Andrew T. (2021). New Insights into Mitochondrial Dysfunction at Disease Susceptibility Loci in the Development of Type 2 Diabetes. Front. Endocrinol..

[67] Hasheminasabgorji E., Jha J.C. (2021). Dyslipidemia, Diabetes and Atherosclerosis: Role of Inflammation and ROS-Redox-Sensitive Factors. Biomedicines.

[68] Wang Z.Q., Jing L.L., Yan J.C., Sun Z., Bao Z.Y., Shao C., Pang Q.W., Geng Y., Zhang L.L., Li L.H. (2018). Role of AGEs in the Progression and Regression of Atherosclerotic Plaques. Glycoconj. J..

[69] Markina Y.V., Gerasimova E.V., Markin A.M., Glanz V.Y., Wu W.K., Sobenin I.A., Orekhov A.N. (2020). Sialylated Immunoglobulins for the Treatment of Immuno-Inflammatory Diseases. Int. J. Mol. Sci..

[70] Bubb K.J., Drummond G.R., Figtree G.A. (2020). New Opportunities for Targeting Redox Dysregulation in Cardiovascular Disease. Cardiovasc. Res..

[71] Ling P., Shan W., Zhai G., Qiu C., Liu Y., Xu Y., Yang X. (2020). Association between Glutathione Peroxidase-3 Activity and Carotid Atherosclerosis in Patients with Type 2 Diabetes Mellitus. Brain Behav..

[72] Yamagishi S., Matsui T. (2018). Role of Hyperglycemia-Induced Advanced Glycation End Product (AGE) Accumulation in Atherosclerosis. Ann. Vasc. Dis..

[73] Laakso M., Kuusisto J. (2014). Insulin Resistance and Hyperglycaemia in Cardiovascular Disease Development. Nat. Rev. Endocrinol..

[74] Lin P., Ji H.H., Li Y.J., Guo S.D. (2021). Macrophage Plasticity and Atherosclerosis Therapy. Front. Mol. Biosci..

[75] Poznyak A., Grechko A.V., Poggio P., Myasoedova V.A., Alfieri V., Orekhov A.N. (2020). The Diabetes Mellitus-Atherosclerosis Connection: The Role of Lipid and Glucose Metabolism and Chronic Inflammation. Int. J. Mol. Sci..

[76] Markin A.M., Markina Y.V., Sukhorukov V.N., Khaylov A.M., Orekhov A.N. (2019). The Role of Physical Activity in the Development of Atherosclerotic Lesions of the Vascular Wall. Clin. Exp. Morphol..

[77] Aberdeen H., Battles K., Taylor A., Garner-Donald J., Davis-Wilson A., Rogers B.T., Cavalier C., Williams E.D. (2021). The Aging Vasculature: Glucose Tolerance, Hypoglycemia and the Role of the Serum Response Factor. J. Cardiovasc. Dev. Dis..

[78] Luna P., Guarner V., Farías J.M., Hernández-Pacheco G., Martínez M. (2016). Importance of Metabolic Memory in the Development of Vascular Complications in Diabetic Patients. J. Cardiothorac. Vasc. Anesth..

[79] Colliva A., Braga L., Giacca M., Zacchigna S. (2020). Endothelial Cell-Cardiomyocyte Crosstalk in Heart Development and Disease. J. Physiol..

[80] Wang M., Li Y., Li S., Lv J. (2022). Endothelial Dysfunction and Diabetic Cardiomyopathy. Front. Endocrinol..

[81] Horton W.B., Barrett E.J. (2021). Microvascular Dysfunction in Diabetes Mellitus and Cardiometabolic Disease. Endocr. Rev..

[82] Salvador D.B., Gamba M.R., Gonzalez-Jaramillo N., Gonzalez-Jaramillo V., Raguindin P.F.N., Minder B., Gräni C., Wilhelm M., Stettler C., Doria A. (2022). Diabetes and Myocardial Fibrosis: A Systematic Review and Meta-Analysis. JACC Cardiovasc. Imaging.

[83] Sinha S., Haque M. (2022). Insulin Resistance Is Cheerfully Hitched with Hypertension. Life.

[84] Palazzuoli A., Iacoviello M. (2022). Diabetes Leading to Heart Failure and Heart Failure Leading to Diabetes: Epidemiological and Clinical Evidence. Heart Fail. Rev..

[85] Chaudhury A., Duvoor C., Reddy Dendi V.S., Kraleti S., Chada A., Ravilla R., Marco A., Shekhawat N.S., Montales M.T., Kuriakose K. (2017). Clinical Review of Antidiabetic Drugs: Implications for Type 2 Diabetes Mellitus Management. Front. Endocrinol..

[86] Padhi S., Nayak A.K., Behera A. (2020). Type II Diabetes Mellitus: A Review on Recent Drug Based Therapeutics. Biomed. Pharmacother. Biomed. Pharmacother..

[87] Drzewoski J., Hanefeld M. (2021). The Current and Potential Therapeutic Use of Metformin—The Good Old Drug. Pharmaceuticals.

[88] Malin S.K., Stewart N.R. (2020). Metformin May Contribute to Inter-Individual Variability for Glycemic Responses to Exercise. Front. Endocrinol..

[89] Sanchez-Rangel E., Inzucchi S.E. (2017). Metformin: Clinical Use in Type 2 Diabetes. Diabetologia.

[90] Tan S.Y., Mei Wong J.L., Sim Y.J., Wong S.S., Mohamed Elhassan S.A., Tan S.H., Ling Lim G.P., Rong Tay N.W., Annan N.C., Bhattamisra S.K. (2019). Type 1 and 2 Diabetes Mellitus: A Review on Current Treatment Approach and Gene Therapy as Potential Intervention. Diabetes Metab. Syndr..

[91] Wehmeier U.F., Piepersberg W. (2004). Biotechnology and Molecular Biology of the α-Glucosidase Inhibitor Acarbose. Appl. Microbiol. Biotechnol..

[92] Gribble F.M., Meek C.L., Reimann F. (2018). Targeted Intestinal Delivery of Incretin Secretagogues-towards New Diabetes and Obesity Therapies. Peptides.

[93] Hossain U., Das A.K., Ghosh S., Sil P.C. (2020). An Overview on the Role of Bioactive α-Glucosidase Inhibitors in Ameliorating Diabetic Complications. Food Chem. Toxicol..

[94] Narita T., Yokoyama H., Yamashita R., Sato T., Hosoba M., Morii T., Fujita H., Tsukiyama K., Yamada Y. (2012). Comparisons of the Effects of 12-Week Administration of Miglitol and Voglibose on the Responses of Plasma Incretins after a Mixed Meal in Japanese Type 2 Diabetic Patients. Diabetes Obes. Metab..

[95] Derosa G., Maffioli P. (2012). α-Glucosidase Inhibitors and Their Use in Clinical Practice. Arch. Med. Sci..

[96] Nauck M.A., Quast D.R., Wefers J., Meier J.J. (2021). GLP-1 Receptor Agonists in the Treatment of Type 2 Diabetes—State-of-the-Art. Mol. Metab..

[97] Htike Z.Z., Zaccardi F., Papamargaritis D., Webb D.R., Khunti K., Davies M.J. (2017). Efficacy and Safety of Glucagon-like Peptide-1 Receptor Agonists in Type 2 Diabetes: A Systematic Review and Mixed-Treatment Comparison Analysis. Diabetes Obes. Metab..

[98] Davies M.J., D’Alessio D.A., Fradkin J., Kernan W.N., Mathieu C., Mingrone G., Rossing P., Tsapas A., Wexler D.J., Buse J.B. (2018). Management of Hyperglycaemia in Type 2 Diabetes, 2018. A Consensus Report by the American Diabetes Association (ADA) and the European Association for the Study of Diabetes (EASD). Diabetologia.

[99] Meier J.J. (2012). GLP-1 Receptor Agonists for Individualized Treatment of Type 2 Diabetes Mellitus. Nat. Rev. Endocrinol..

[100] Drucker D.J., Buse J.B., Taylor K., Kendall D.M., Trautmann M., Zhuang D., Porter L. (2008). Exenatide Once Weekly versus Twice Daily for the Treatment of Type 2 Diabetes: A Randomised, Open-Label, Non-Inferiority Study. Lancet.

[101] Uppal S., Italiya K.S., Chitkara D., Mittal A. (2018). Nanoparticulate-Based Drug Delivery Systems for Small Molecule Anti-Diabetic Drugs: An Emerging Paradigm for Effective Therapy. Acta Biomater..

[102] Nauck M.A., Meier J.J. (2019). Management of endocrine disease: Are All GLP-1 Agonists Equal in the Treatment of Type 2 Diabetes?. Eur. J. Endocrinol..

[103] Alharby H., Abdelati T., Rizk M., Youssef E., Gaber N., Moghazy K., Yafei S. (2019). Association of Fasting Glucagon-like Peptide-1 with Oxidative Stress and Subclinical Atherosclerosis in Type 2 Diabetes. Diabetes Metab. Syndr..

[104] Crepaldi G., Carruba M., Comaschi M., del Prato S., Frajese G., Paolisso G. (2007). Dipeptidyl Peptidase 4 (DPP-4) Inhibitors and Their Role in Type 2 Diabetes Management. J. Endocrinol. Investig..

[105] Ahrén B., Schmitz O. (2004). GLP-1 Receptor Agonists and DPP-4 Inhibitors in the Treatment of Type 2 Diabetes. Horm. Metab. Res. Horm. Und Stoffwechs. Horm. Metab..

[106] Deacon C.F., Ahrén B., Holst J.J. (2004). Inhibitors of Dipeptidyl Peptidase IV: A Novel Approach for the Prevention and Treatment of Type 2 Diabetes?. Expert Opin. Investig. Drugs.

[107] Esposito K., Chiodini P., Maiorino M.I., Bellastella G., Capuano A., Giugliano D. (2014). Glycaemic Durability with Dipeptidyl Peptidase-4 Inhibitors in Type 2 Diabetes: A Systematic Review and Meta-Analysis of Long-Term Randomised Controlled Trials. BMJ Open.

[108] Aroda V.R., Henry R.R., Han J., Huang W., DeYoung M.B., Darsow T., Hoogwerf B.J. (2012). Efficacy of GLP-1 Receptor Agonists and DPP-4 Inhibitors: Meta-Analysis and Systematic Review. Clin. Ther..

[109] Samms R.J., Christe M.E., Collins K.A.L., Pirro V., Droz B.A., Holland A.K., Friedrich J.L., Wojnicki S., Konkol D.L., Cosgrove R. (2021). GIPR Agonism Mediates Weight-Independent Insulin Sensitization by Tirzepatide in Obese Mice. J. Clin. Investig..

[110] Campbell J.E. (2021). Targeting the GIPR for Obesity: To Agonize or Antagonize? Potential Mechanisms. Mol. Metab..

[111] Matza L.S., Stewart K.D., Landó L.F., Patel H., Boye K.S. (2022). Exit Interviews Examining the Patient Experience in Clinical Trials of Tirzepatide for Treatment of Type 2 Diabetes. Patient.

[112] Nauck M.A., Quast D.R., Wefers J., Pfeiffer A.F.H. (2021). The Evolving Story of Incretins (GIP and GLP-1) in Metabolic and Cardiovascular Disease: A Pathophysiological Update. Diabetes Obes. Metab..

[113] Thangavel N., al Bratty M., Javed S.A., Ahsan W., Alhazmi H.A. (2017). Targeting Peroxisome Proliferator-Activated Receptors Using Thiazolidinediones: Strategy for Design of Novel Antidiabetic Drugs. Int. J. Med. Chem..

[114] Kroker A.J., Bruning J.B. (2015). Review of the Structural and Dynamic Mechanisms of PPARγ Partial Agonism. PPAR Res..

[115] Grygiel-Górniak B. (2014). Peroxisome Proliferator-Activated Receptors and Their Ligands: Nutritional and Clinical Implications—A Review. Nutr. J..

[116] Lamichane S., Lamichane B.D., Kwon S.M. (2018). Pivotal Roles of Peroxisome Proliferator-Activated Receptors (PPARs) and Their Signal Cascade for Cellular and Whole-Body Energy Homeostasis. Int. J. Mol. Sci..

[117] Zoete V., Grosdidier A., Michielin O. (2007). Peroxisome Proliferator-Activated Receptor Structures: Ligand Specificity, Molecular Switch and Interactions with Regulators. Biochim. Biophys. Acta BBA Mol. Cell Biol. Lipids.

[118] Lebovitz H.E. (2019). Thiazolidinediones: The Forgotten Diabetes Medications. Curr. Diabetes Rep..

[119] Hsueh W.A., Law R. (2003). The Central Role of Fat and Effect of Peroxisome Proliferator-Activated Receptor–γ on Progression of Insulin Resistance and Cardiovascular Disease. Am. J. Cardiol..

[120] Ivanova E.A., Parolari A., Myasoedova V., Melnichenko A.A., Bobryshev Y.V., Orekhov A.N. (2015). Peroxisome Proliferator-Activated Receptor (PPAR) Gamma in Cardiovascular Disorders and Cardiovascular Surgery. J. Cardiol..

[121] Ricote M., Glass C.K. (2007). PPARs and Molecular Mechanisms of Transrepression. Biochim. Biophys. Acta.

[122] Krishnaswami A., Ravi-Kumar S., Lewis J.M. (2010). Thiazolidinediones: A 2010 Perspective. Perm. J..

[123] McGuire D.K., Inzucchi S.E. (2008). New Drugs for the Treatment of Diabetes Mellitus: Part I: Thiazolidinediones and Their Evolving Cardiovascular Implications. Circulation.

[124] Stumvoll M., Goldstein B.J., van Haeften T.W. (2005). Type 2 Diabetes: Principles of Pathogenesis and Therapy. Lancet.

[125] Ahmed I., Furlong K., Flood J., Treat V.P., Goldstein B.J. (2007). Dual PPAR Alpha/Gamma Agonists: Promises and Pitfalls in Type 2 Diabetes. Am. J. Ther..

[126] Mirza A.Z., Althagafi I.I., Shamshad H. (2019). Role of PPAR Receptor in Different Diseases and Their Ligands: Physiological Importance and Clinical Implications. Eur. J. Med. Chem..

[127] Yamasaki Y., Katakami N., Furukado S., Kitagawa K., Nagatsuka K., Kashiwagi A., Daida H., Kawamori R., Kaku K. (2010). Long-Term Effects of Pioglitazone on Carotid Atherosclerosis in Japanese Patients with Type 2 Diabetes without a Recent History of Macrovascular Morbidity. J. Atheroscler. Thromb..

[128] Saremi A., Schwenke D.C., Buchanan T.A., Hodis H.N., MacK W.J., Banerji M., Bray G.A., Clement S.C., Henry R.R., Kitabchi A.E. (2013). Pioglitazone Slows Progression of Atherosclerosis in Prediabetes Independent of Changes in Cardiovascular Risk Factors. Arter. Thromb Vasc. Biol..

[129] Tilinca M.C., Tiuca R.A., Tilea I., Varga A. (2021). The Sglt-2 Inhibitors in Personalized Therapy of Diabetes Mellitus Patients. J. Pers. Med..

[130] Hsia D.S., Grove O., Cefalu W.T. (2017). An Update on Sodium-Glucose Co-Transporter-2 Inhibitors for the Treatment of Diabetes Mellitus. Curr. Opin. Endocrinol. Diabetes Obes..

[131] Brown E., Rajeev S.P., Cuthbertson D.J., Wilding J.P.H. (2019). A Review of the Mechanism of Action, Metabolic Profile and Haemodynamic Effects of Sodium-Glucose Co-Transporter-2 Inhibitors. Diabetes Obes. Metab..

[132] Bhatt D.L., Szarek M., Steg P.G., Cannon C.P., Leiter L.A., McGuire D.K., Lewis J.B., Riddle M.C., Voors A.A., Metra M. (2021). Sotagliflozin in Patients with Diabetes and Recent Worsening Heart Failure. N. Engl. J. Med..

[133] Pereira M.J., Eriksson J.W. (2019). Emerging Role of SGLT-2 Inhibitors for the Treatment of Obesity. Drugs.

[134] Scheen A.J. (2015). Pharmacodynamics, Efficacy and Safety of Sodium-Glucose Co-Transporter Type 2 (SGLT2) Inhibitors for the Treatment of Type 2 Diabetes Mellitus. Drugs.

[135] Hasan F.M., Alsahli M., Gerich J.E. (2014). SGLT2 Inhibitors in the Treatment of Type 2 Diabetes. Diabetes Res. Clin. Pract..

[136] Lambers Heerspink H.J., de Zeeuw D., Wie L., Leslie B., List J. (2013). Dapagliflozin a Glucose-Regulating Drug with Diuretic Properties in Subjects with Type 2 Diabetes. Diabetes Obes. Metab..

[137] Sjöström C.D., Hashemi M., Sugg J., Ptaszynska A., Johnsson E. (2015). Dapagliflozin-Induced Weight Loss Affects 24-Week Glycated Haemoglobin and Blood Pressure Levels. Diabetes Obes. Metab..

[138] Tikkanen I., Narko K., Zeller C., Green A., Salsali A., Broedl U.C., Woerle H.J. (2015). Empagliflozin Reduces Blood Pressure in Patients with Type 2 Diabetes and Hypertension. Diabetes Care.

[139] Kaplan A., Abidi E., El-Yazbi A., Eid A., Booz G.W., Zouein F.A. (2018). Direct Cardiovascular Impact of SGLT2 Inhibitors: Mechanisms and Effects. Heart Fail. Rev..

[140] Ojima A., Matsui T., Nishino Y., Nakamura N., Yamagishi S. (2015). Empagliflozin, an Inhibitor of Sodium-Glucose Cotransporter 2 Exerts Anti-Inflammatory and Antifibrotic Effects on Experimental Diabetic Nephropathy Partly by Suppressing AGEs-Receptor Axis. Horm. Metab. Res..

[141] Kothari V., Galdo J.A., Mathews S.T. (2016). Hypoglycemic Agents and Potential Anti-Inflammatory Activity. J. Inflamm. Res..

[142] Kim J., Park J.H., Shah K., Mitchell S.J., Cho K., Hoe H.S. (2021). The Anti-Diabetic Drug Gliquidone Modulates Lipopolysaccharide-Mediated Microglial Neuroinflammatory Responses by Inhibiting the NLRP3 Inflammasome. Front. Aging Neurosci..

[143] Jahan H., Choudhary M.I. (2021). Gliclazide Alters Macrophages Polarization State in Diabetic Atherosclerosis in Vitro via Blocking AGE-RAGE/TLR4-Reactive Oxygen Species-Activated NF-Kβ Nexus. Eur. J. Pharm..

[144] Postler T.S., Peng V., Bhatt D.M., Ghosh S. (2021). Metformin Selectively Dampens the Acute Inflammatory Response through an AMPK-Dependent Mechanism. Sci. Rep..

[145] Kristófi R., Eriksson J.W. (2021). Metformin as an Anti-Inflammatory Agent: A Short Review. J. Endocrinol..

[146] Zangiabadian M., Nejadghaderi S.A., Zahmatkesh M.M., Hajikhani B., Mirsaeidi M., Nasiri M.J. (2021). The Efficacy and Potential Mechanisms of Metformin in the Treatment of COVID-19 in the Diabetics: A Systematic Review. Front. Endocrinol..

[147] Bassaganya-Riera J., Song R., Roberts P.C., Hontecillas R. (2010). PPAR-Gamma Activation as an Anti-Inflammatory Therapy for Respiratory Virus Infections. Viral Immunol..

[148] Klimcakova E., Moro C., Mazzucotelli A., Lolmède K., Viguerie N., Galitzky J., Stich V., Langin D. (2007). Profiling of Adipokines Secreted from Human Subcutaneous Adipose Tissue in Response to PPAR Agonists. Biochem. Biophys. Res. Commun..

[149] Della Pepa G., Russo M., Vitale M., Carli F., Vetrani C., Masulli M., Riccardi G., Vaccaro O., Gastaldelli A., Rivellese A.A. (2021). Pioglitazone Even at Low Dosage Improves NAFLD in Type 2 Diabetes: Clinical and Pathophysiological Insights from a Subgroup of the TOSCA.IT Randomised Trial. Diabetes Res. Clin. Pract..

[150] Wu W., Liu L., Zhu H., Sun Y., Wu Y., Liao H., Gui Y., Li L., Liu L., Sun F. (2019). Butyrolactone-I, an Efficient α-Glucosidase Inhibitor, Improves Type 2 Diabetes with Potent TNF-α-Lowering Properties through Modulating Gut Microbiota in Db/Db Mice. FASEB J..

[151] Emoto T., Sawada T., Hashimoto M., Kageyama H., Terashita D., Mizoguchi T., Mizuguchi T., Motodi Y., Iwasaki M., Taira K. (2012). Effect of 3-Month Repeated Administration of Miglitol on Vascular Endothelial Function in Patients with Diabetes Mellitus and Coronary Artery Disease. Am. J. Cardiol..

[152] Tahara A., Kurosaki E., Yokono M., Yamajuku D., Kihara R., Hayashizaki Y., Takasu T., Imamura M., Li Q., Tomiyama H. (2014). Effects of Sodium-Glucose Cotransporter 2 Selective Inhibitor Ipragliflozin on Hyperglycaemia, Oxidative Stress, Inflammation and Liver Injury in Streptozotocin-Induced Type 1 Diabetic Rats. J. Pharm. Pharm..

[153] Pawlos A., Broncel M., Woźniak E., Gorzelak-Pabiś P. (2021). Neuroprotective Effect of SGLT2 Inhibitors. Molecules.

[154] Lee Y.S., Park M.S., Choung J.S., Kim S.S., Oh H.H., Choi C.S., Ha S.Y., Kang Y., Kim Y., Jun H.S. (2012). Glucagon-like Peptide-1 Inhibits Adipose Tissue Macrophage Infiltration and Inflammation in an Obese Mouse Model of Diabetes. Diabetologia.

[155] Wang X.C., Gusdon A.M., Liu H., Qu S. (2014). Effects of Glucagon-like Peptide-1 Receptor Agonists on Non-Alcoholic Fatty Liver Disease and Inflammation. World J. Gastroenterol..

[156] Hirakawa H., Zempo H., Ogawa M., Watanabe R., Suzuki J.I., Akazawa H., Komuro I., Isobe M. (2015). A DPP-4 Inhibitor Suppresses Fibrosis and Inflammation on Experimental Autoimmune Myocarditis in Mice. PLoS ONE.

[157] Dai Y., Dai D., Wang X., Ding Z., Mehta J.L. (2014). DPP-4 Inhibitors Repress NLRP3 Inflammasome and Interleukin-1beta via GLP-1 Receptor in Macrophages through Protein Kinase C Pathway. Cardiovasc. Drugs Ther..

[158] Shinjo T., Nakatsu Y., Iwashita M., Sano T., Sakoda H., Ishihara H., Kushiyama A., Fujishiro M., Fukushima T., Tsuchiya Y. (2015). DPP-IV Inhibitor Anagliptin Exerts Anti-Inflammatory Effects on Macrophages, Adipocytes, and Mouse Livers by Suppressing NF-ΚB Activation. Am. J. Physiol. Endocrinol. Metab..

